# Indolic Uremic Solutes Enhance Procoagulant Activity of Red Blood Cells through Phosphatidylserine Exposure and Microparticle Release

**DOI:** 10.3390/toxins7114390

**Published:** 2015-10-28

**Authors:** Chunyan Gao, Shuting Ji, Weijun Dong, Yushan Qi, Wen Song, Debin Cui, Jialan Shi

**Affiliations:** 1 Department of Medical Laboratory Science and Technology, Harbin Medical University-Daqing, 39 Xinyang Road, Gaoxin District, Daqing 163319, China; E-Mails: jishuting@tom.com (S.J.); jianyanzhongxin@126.com (Y.Q.); fyzqx@sina.com (W.S); cdb1024@163.com (D.C.); 2 Department of General Surgery, The Fifth Hospital; Harbin Medical University-Daqing, 39 Xinyang Road, Gaoxin District, Daqing 163319, China; E-Mail: dongweijun19771977@163.com; 3 Department of Hematology, The First Hospital, Harbin Medical University, 23 Youzheng Street, Nangang District, Harbin 150001, China; 4 Department of Medicine, Brigham and Women’s Hospital, Harvard Medical School, Boston, MA 02132, USA

**Keywords:** indoxyl sulfate, indole-3-acetic acid, phosphatidylserine, microparticle, procoagulant, red blood cell

## Abstract

Increased accumulation of indolic uremic solutes in the blood of uremic patients contributes to the risk of thrombotic events. Red blood cells (RBCs), the most abundant blood cells in circulation, may be a privileged target of these solutes. However, the effect of uremic solutes indoxyl sulfate (IS) and indole-3-acetic acid (IAA) on procoagulant activity (PCA) of erythrocyte is unclear. Here, RBCs from healthy adults were treated with IS and IAA (mean and maximal concentrations reported in uremic patients). Phosphatidylserine (PS) exposure of RBCs and their microparticles (MPs) release were labeled with Alexa Fluor 488-lactadherin and detected by flow cytometer. Cytosolic Ca^2+^ ([Ca^2+^]) with Fluo 3/AM was analyzed by flow cytometer. PCA was assessed by clotting time and purified coagulation complex assays. We found that PS exposure, MPs generation, and consequent PCA of RBCs at mean concentrations of IS and IAA enhanced and peaked in maximal uremic concentrations. Moreover, 128 nM lactadherin, a PS inhibitor, inhibited over 90% PCA of RBCs and RMPs. Eryptosis or damage, by indolic uremic solutes was due to, at least partially, the increase of cytosolic [Ca^2+^]. Our results suggest that RBC eryptosis in uremic solutes IS and IAA plays an important role in thrombus formation through releasing RMPs and exposing PS. Lactadherin acts as an efficient anticoagulant in this process.

## 1. Introduction

Uremia is characterized by the retention of several metabolites (collectively termed here as uremic solutes or uremic toxins). Based on the ability of these solutes to undergo clearance with dialysis, they are classified into three groups: small water-soluble molecules, middle molecules, and protein-bound solutes [1]. Indoxyl sulfate (IS) and indoxyl-3-acetate acid (IAA), two of protein-bound solutes, which are tryptophan metabolites derived from indole, are poorly removed by conventional dialysis and present a particular risk for uremic thrombophilia and accelerated atherosclerosis [2].

Recent studies have revealed that indolic solutes (IS and IAA) may enhance thrombosis through induce tissue factor (TF) in both endothelial cells (ECs) and vascular smooth muscle cells (vSMCs) [3,4]. However, as the most abundant cells in circulating blood, red blood cells (RBCs) are not innocent bystanders of hemostasis and thrombosis [5,6]. They are in permanent contact with uremic retention solutes and could be a privileged target of these solutes. The effects of indolic solutes, specifically on procoagulant activity (PCA) of RBC are still not well understood.

Phosphatidylserine (PS), an anionic phospholipid, is usually confined to the inner leaflet of the cell membrane [7]. During cell injury, apoptosis, or activation, PS is externalized to the outer membrane and accompanied with vesicles release, also called microparticles (MPs), which expose PS and express membrane antigens on their surfaces [8,9]. Moreover, under the stimulation of anticarcinogenic drugs such as PRIMA-1 and alantolactone *et al.*, RBC may enter suicidal death or eryptosis, which is characterized by cell shrinkage and cell membrane scrambling with PS exposure at the RBC surface [10,11]. PS is necessary for assembly of tenase and prothrombinase that results in thrombin generation [12]. Lang *et al.* (2013) reported that IS can induce PS translocation to the external surface of the RBC membrane [13], however, the implications of PS-externalized RBCs in PCA and subsequent thrombus formation were not addressed. Therefore, we investigated whether PS on RBC and RBC-derived MPs (RMPs) contributes to indolic solutes-associated thrombophilia.

Lactadherin, a sensitive probe for PS exposure and an anticoagulant by competing with factors V and VIII for PS-containing membranes, is independent of Ca^2+^ and membrane PE content [14,15,16,17]. In the present study, lactadherin was used as a novel probe for the detection of PS exposure and RMPs release, and an anticoagulant for inhibition of the PCA of RBC in this study.

In this study, an integrated assessment of indolic solutes (IS and IAA) enhanced thrombogenicity was performed. We measured the levels of PS exposure and MP release on RBC by mimicking mean and maximal concentrations of IS and IAA reported in uremic patients. Of note, we examined the PS-dependent contribution of RMPs and RBC to thrombosis formation.

## 2. Results and Discussion

### 2.1. IS and IAA Induced PS Exposure and MPs Release of RBCs

**Figure 1:**
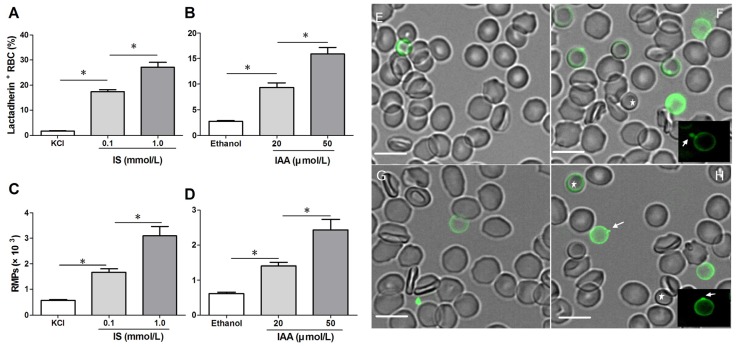
Detection of phosphatidylserine (PS) exposure and microparticles (MPs) release on red blood cells (RBCs). RBCs from healthy volunteers were incubated with median and maximal uremic concentration of IS and IAA for 24 h, respectively. KCl or ethanol was utilized as their respective controls. (**A**,**B**) Lactadherin-binding percent of RBCs was evaluated by flow cytometry. Results represent the mean ± SD of four independent experiments (* *p* < 0.001). (**C**,**D**) After indolic uremic solutes treatment for 24 h, MPs from 10 mL of the RBCs supernatants was harvested, and stained with Alexa Fluor 488-lactadherin and Alexa Fluor 647-CD235a. RMPs were defined as smaller than 1 μm and coexpression of lactadherin and CD235a. The number of RMP per μL culture medium was examined using flow cytometry. Results represent the mean ± SD of four independent experiments (* *p* < 0.001). PS exposure and MPs release on RBCs using confocal microscopy. RBCs were stained with Alexa Fluor 488-lactadherin in the dark at room temperature. RBC membrane displayed green fluorescence when labelled by Alexa Fluor 488-lactadherin. Few lactadherin staining was observed on RBCs cultured in KCl (**E**) or ethanol (**G**). Treatment of RBCs with 1.0 mM IS (**F**) or 50 μM IAA (**H**) for 24 h led to PS externalized to the outer membrane, vesicles released from the budding of cellular membranes. Arrows indicate MP generation (appeared green) on erythrocyte membranes, and the stars indicate spherocyte. Bars represent 10 μm. PS, phosphatidylserine; IS, Indoxyl sulfate; IAA, indoxyl-3-acetate acid; RBC, red blood cell, MPs, microparticles; RMPs, RBC derived MPs.

To confirm RBC damage or eryptosis in IS and IAA, we utilized Alexa Fluor 488-lactadherin to detect PS exposure on RBCs and RMPs release by flow cytometer. After 24 h of incubation at the mean and maximal concentrations of IS and IAA found in chronic kidney disease (CKD) patients, we found that the percentage of lactadherin^+^ RBC in IS (0.1 mM) was significantly higher than that in control (*p* < 0.001), with more lactadherin^+^ RBC in maximal levels of IS (1 mM) than that in median levels of IS (0.1 mM) (*p* < 0.001). IAA also induced a significant increase in PS exposure after 24 h of incubation (*p* < 0.001 *vs.* control), and which paralleled the increasing IAA concentrations (Figure 1A,B). In another time-response experiment, IS (1 mM) or IAA (50 μM) induced a significant increase in PS exposure after 4 h of incubation, and increased rapidly during the first 24 h. From 24 h to 48 h, the proportion of lactadherin^+^ RBC continued to increase slowly, while a control group was almost unchanged during 48 h (Figure S1).

At the same time, we analyzed RMPs shedding by flow cytometry. The level of RMPs obtained with IS or IAA is higher than those obtained with control (KCl for IS, ethanol for IAA), and these levels paralleled with uremic solutes concentrate (Figure 1C,D).

To visually identify PS on the outer membrane of RBCs and RMPs generation, RBCs were incubated with Alexa Fluor 488-lactadherin and imaged on a confocal microscopy. As shown in Figure 1F,H, partly RBCs exhibited volume reduction from normal discocytic shapes and changed into spherocytes with strong green fluorescence stained by lactadherin on the outer membrane and accompanying vesiculation formation. IS or IAA treatment induced typical MPs generation (the arrows indicate RMPs). Only very little staining by fluorescence-labeled lactadherin could be detected on control RBC (Figure 1E,G), which is in agreement with the findings presented in the flow cytometry measurements.

### 2.2. Role of Indolic Uremic Solutes on Erythrocyte Cytosolic Ca^2+^ Concentration

**Figure 2:**
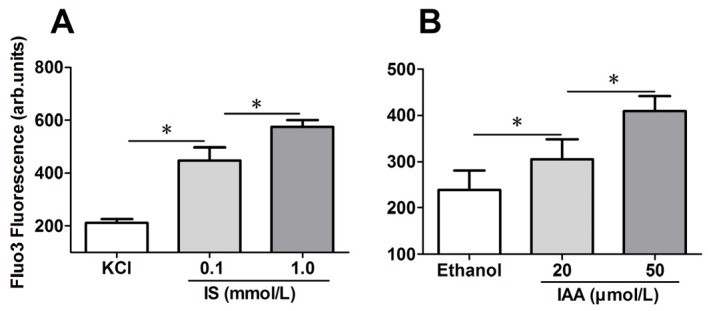
Role of uremic solution on erythrocyte cytosolic Ca^2+^ concentration. Following exposure for 24 h to the different indicated concentration of indolic uremic solutes, erythrocytes were washed in Ringer solution and then loaded with Fluo 3/AM. Ca^2+^ dependent fluorescence intensity was measured with flow cytometry. The Fluo-3 mean fluorescence intensity (MFI) (arbitrary units) in erythrocytes exposed to culture media with indoxyl sulfate (IS) (**A**) and indoxyl-3-acetate acid (IAA) (**B**) was shown. Data are displayed as mean ± SD for triplicate samples of independent experiments, * indicate *p* < 0.001.

To explore the mechanism responsible for the PS exposure of RBCs in indolic uremic solutes, further experiments were performed to test cytosolic Ca^2+^ concentration ([Ca^2+^]) using flow cytometry and results are represented by Ca^2+^ dependent fluorescence intensity. As illustrated in Figure 2A,B, in comparison to that in control conditions, median levels of IS and IAA induced a significant increase of erythrocyte cytosolic [Ca^2+^] after 24 h of incubation. Furthermore, there was a significant increase of [Ca^2+^] in maximal levels of IS (1 mM) and IAA (50 μM) than that in median uremic concentration, respectively (*p* < 0.001).

### 2.3. Effect of Ca^2+^ Withdrawal on IS and IAA Induced PS Exposure

**Figure 3:**
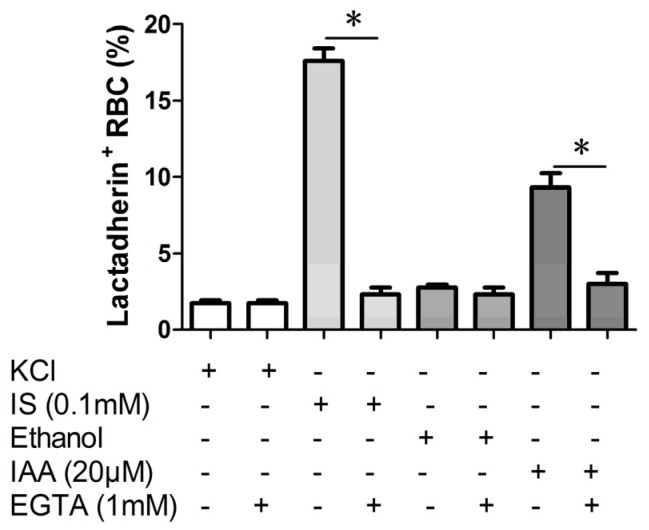
Effect of Ca^2+^ withdrawal on IS and IAA induced PS exposure. The percentage of lactadherin binding erythrocytes after a 24 h treatment with IS (0.1 mM) or IAA (20 μM) in the presence and absence of EGTA (1 mM). KCl or ethanol was utilized as their respective controls. Data are displayed as mean ± SD for triplicate samples of independent experiments, * indicate *p* < 0.001.

In order to verify whether the PS exposure on erythrocyte by indolic uremic solutes was secondary to an increase of [Ca^2+^], erythrocytes were exposed to IS (0.1 mM) and IAA (20 μM) for 24 h either in the presence of extracellular Ca^2+^ (1 mM) or in the nominal absence of Ca^2+^ and presence of the Ca^2+^ chelator EGTA (1 mM). As shown in Figure 3, the effect of IS and IAA on the increased PS exposure was virtually abolished in the absence of Ca^2+^.

### 2.4. IS and IAA Increase the PCA of RBCs

**Figure 4:**
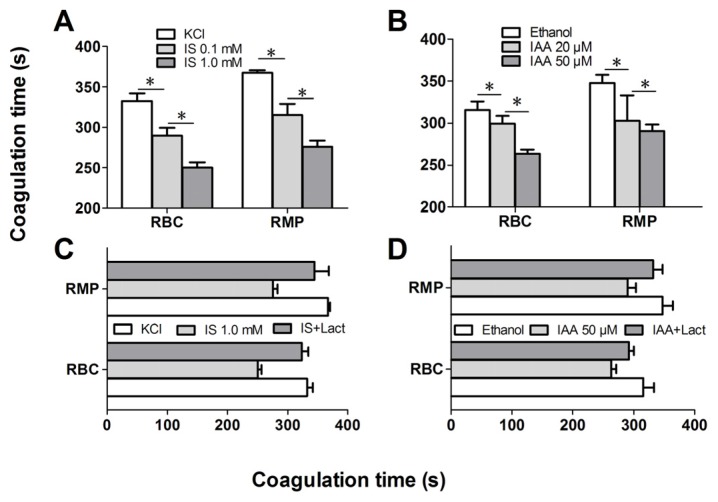
Recalcification time and inhibition assay. RBCs were treated with different concentrations of IS or IAA for 24 h, cells and RMPs were collected, respectively. KCl or ethanol was utilized as their respective controls. Coagulation times of 100 μL RBCs (1 × 10^8^) and RMPs (prepared from 10 mL of the RBCs supernatants) in each group of IS (**A**) and IAA (**B**) are shown. PCA of RBCs and RMPs that incubated in IS (**C**) and IAA (**D**) were detected in the absence or presence of 128 nM lactadherin. Data are displayed as mean ± SD for triplicate samples of independent experiments, * indicate *p* < 0.001.

Further experiments explored whether indolic uremic solutes triggered PCA of RBCs. We first evaluated the PCA of RBCs and RMPs by recalcification-time assays in the absence or presence of IS and IAA. Incremental PCA was exhibited by reductive clotting time. As shown in Figure 4A,B, the coagulation time was significantly reduced in IS (0.1 mM) and IAA (20 μM) compared with RBCs in control medium (KCl for IS, ethanol for IAA) after 24 h of incubation (*p* < 0.001), with shorter coagulation time in maximal levels of IS (1 mM) and IAA (50 μM) than in median uremic concentration (*p* < 0.001). For RMPs, IS and IAA enhanced the PCA also in a concentration-dependent fashion. The reduction of coagulation time was in accord with the number of RMPs. In order to explore the relationship between PS exposure and PCA of RBCs that induced by IS or IAA, we performed coagulation inhibition assays. PCA of both RBCs and RMPs were almost completely inhibited by 128 nM lactadherin, as described in Figure 4C,D.

**Figure 5:**
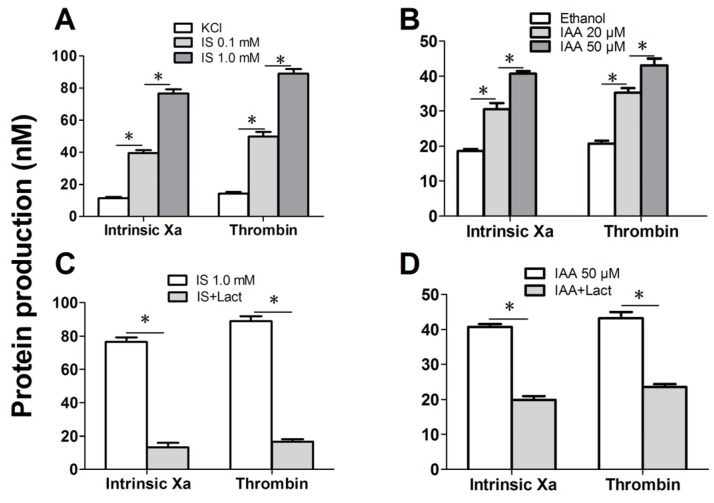
Formation and inhibition assays of procoagulant enzyme complexes. After exposure for 24 h to the different indicated concentration of IS or IAA or KCl (control) or ethanol (control), erythrocytes were washed with Ringer solution. Intrinsic FXa formation was measured in the presence of FIXa, FVIII and thrombin. Thrombin generation was investigated in the presence of FXa and FVa. Intrinsic FXa and thrombin production of 10^5^ RBCs in each group of IS (**A**) and IAA (**B**) are shown. The capacity of 128 nM lactadherin to block procoagulant enzyme complexes on RBCs that incubated in IS (**C**) and IAA (**D**) was evaluated. Results represent the mean ± SD for triplicate of independent experiments, * indicate *p* < 0.001.

We further investigated the capacity of RBCs to support intrinsic FXa and thrombin that contribute to PCA. As depicted in Figure 5A, the production of the two procoagulant enzyme complexes was increased in median uremic concentration of IS (0.1 mM) compared with controls (*p* < 0.001), and more higher in maximal levels of IS (1 mM). Similar results were obtained on IAA treated RBC, the rise of thrombin and intrinsic FXa production paralleled the increasing IAA concentration (Figure 5B). Inhibition assays of FXa and prothrombinase productions were also performed. Over 90% of the production of two procoagulant enzyme complexes was inhibited by 128 nM lactadherin (Figure 5C,D). Results from inhibition assays confirmed further that PS played a crucial role in the PCA of indolic uremic solutes treated RBCs.

**Figure 6:**
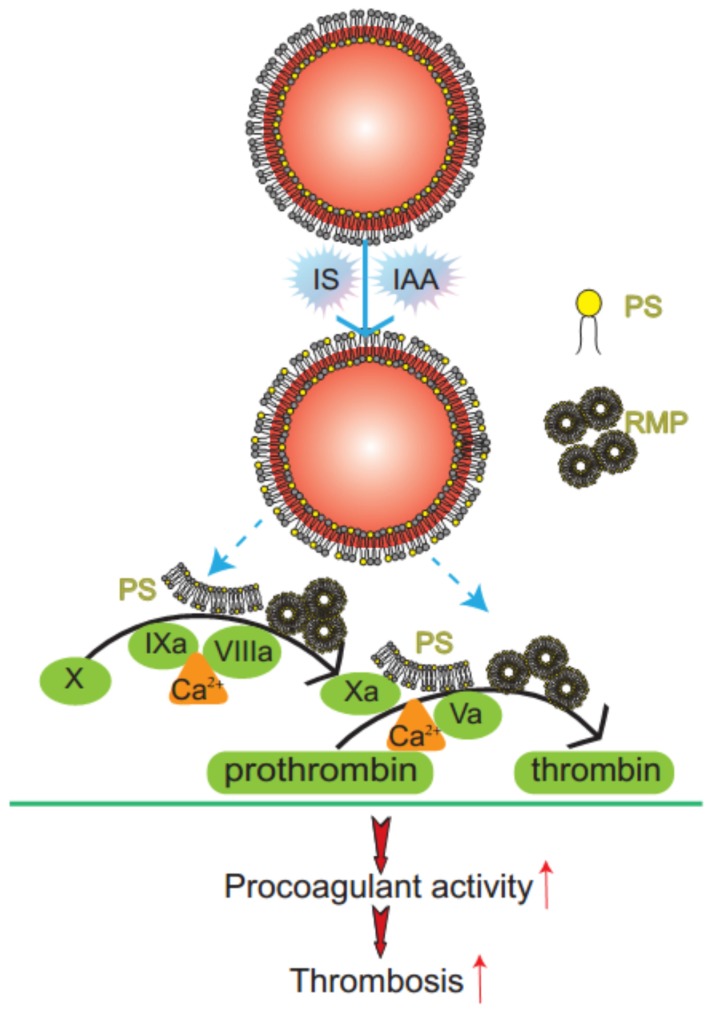
Mechanism diagram of indolic solutes-induced PCA of erythrocytes. IS and IAA triggered PS externalized on the outer membrane of RBC and accompanied with RMPs shedding. Externalized PS on RBCs and PS-bearing RMPs provided binding sites for FXa and prothrombinase complexes, increased thrombin production and enhanced thrombus formation. PS, phosphatidylserine; RMPs, RBC derived MPs; IS, Indoxyl sulfate; IAA, indoxyl-3-acetate acid; PCA, procoagulant activity.

### 2.5. Discussion

In this study, we have found that indolic uremic solutes (IS or IAA) induced a procoagulant phenotype on RBC through increased PS exposure and RMP release. Exposed PS of RBCs and RMPs supports the assembly of intrinsic FXa and prothrombinase, moreover, blockade of PS with lactadherin inhibits activity of procoagulant enzyme complexes and consequently decreases the PCA of RBCs and RMPs (Figure 6). Furthermore, we showed that PS translocation to the RBC surface within indolic uremic solutes is at least partially due to increased cytosolic Ca^2+^ concentration.

The protein-bound uremic solutes IS and IAA are poorly removed by conventional dialysis and probably contribute to cardiovascular risk during the progression of chronic kidney disease (CKD) [18]. RBCs could be a major concern because they make up more than 99% of the total blood cells and live for about 120 days in the circulation system and could be particularly deleterious in these uremic solutes. However, the role of erythrocyte in indolic uremic solutes-associated hemostatic disorders has not been fully evaluated. RBC do not express tissue factor [19]. We therefore designed this study to evaluate the effects of IS and IAA on PS exposure, MPs release of RBCs and consequent PCA.

Our results showed that IS and IAA were able to induce the PS externalization and RMPs release of RBC. Compared with the results of Lang *et al.* [13], more higher levels of PS exposure on RBC was detected by Alexa Fluor 488-lactadherin, because lactadherin is more sensitive for detecting PS exposure than annexin V [16,20]. Unlike annexin V, the lactadherin assay is Ca^2+^ independent, making it suitable for our detection of PS exposure on RBC in the absence of extracelluar calcium. Under confocal microscopy using Alexa Fluor 488-lactadherin, we visually identify PS externalization on erythrocyte membranes accompanying MPs formation in uremic solutes. These erythrocytes change into spherocytes by a substantial loss of membrane surface through vesiculation and MPs generation. All these results reveal that IS and IAA trigger a suicidal erythrocyte death that is eryptosis [21,22].

The role of erythrocyte in indolic uremic solutes-associated hemostatic disorders has not been fully evaluated. Our prior research found that RBC is involved in hypercoagulability in nephrotic syndrome (NS) and polycythemia vera (PV) via PS exposure and MPs shedding [23,24]. In this study, we evaluated the prothrombotic impact of IS and IAA on RBCs. We found that the increased RMPs shedding and exposure of PS on the surface of RBCs provides binding sites for FXa and prothrombinase complexes, thus promoting the coagulation cascade reaction and subsequently leading to a dramatic increase in thrombin generation. In another experiment, RBCs were pre-incubated with 128 nM lactadherin, which inhibited over 90% production of the FXa and thrombin, and almost restored coagulation times of RBCs and RMPs to control levels. Therefore, strong evidence demonstrates that the PCA is mostly due to exposed PS on the surface of RBCs and shed RMPs. Our present study not only indicated increase of PS exposure and MPs release of RBC in indolic uremic solute but also confirmed one mechanism of IS- and IAA-induced hypercoagulable state, which may be a factor inducing cardiovascular events in CKD. In the present study, we examined only IS and IAA, which are known to have vascular effects, rather than each kind of toxin. Further studies will be necessary to investigate the role of other uremic solutes on RBC.

As eryptosis is triggered by the increase of cytosolic Ca^2+^ concentration ([Ca^2+^]) [25,26,27], fluo3 fluorescence was employed to estimate [Ca^2+^]. Our results showed that cytosolic [Ca^2+^] was increased significantly in indolic uremic solutes and associated with increased PS exposure and RMPs release. Upon the increase of cytosolic [Ca^2+^], scramblase presumably mediate PS translocation to the erythrocyte surface with membrane blebbing and MPs shedding [28,29]. Removal of extracellular Ca^2+^ significantly blunted the effect of IS and IAA on lactadherin binding. Our study shows that the increased cytosolic [Ca^2+^] might contribute to IS and IAA-induced PS exposure and MPs generation in erythrocyte. The underlying molecular mechanism will be required to define our future studies.

Lactadherin, an effective PS-stained reagent was used as a novel probe to quantify and localize PS exposure on RBC. Moreover, lactadherin acts as an effective anticoagulant for inhibiting PCA of IS- and IAA-cultured RBC by blocked PS, which may prevent indolic uremic solutes induced thrombophilia.

In conclusion, we provide new insights into the roles of RBCs in the development of IS and IAA-related hypercoagulable states. We demonstrated that indolic uremic solutes increased PCA of RBC by inducing PS exposure and RMPs release, and the effect is at least partially due to increased cytosolic [Ca^2+^]. Alternatively, it is necessary to further study the possibility of applying lactadherin in thrombotic complications of uremia.

## 3. Materials and Methods

### 3.1. Reagents

Indoxyl sulfate (IS), indole-3-acetic acid (IAA) and EGTA were obtained from Sigma (Saint Quentin Fallavier, France). Megamix beads (mix of 0.5, 0.9 and 3.0 μm beads) were from Biocytex (Marseille, France). Trucount Tube (Cat. No. 340334), Purified CD235a (clone GA-R2) and mouse IgG1/IgG2a (clone X40/X39) were from Becton Dickinson Biosciences (San Jose, CA, USA), and were labeled in our laboratory with Alexa Fluor 647 (Invitrogen, Eugene, OR, USA). Bovine lactadherin was purified as described previously Hvarregaard *et al*. (1996) [30] and were labeled in our laboratory with Alexa Fluor 488 (Invitrogen). Fluo 3/AM was from Biotium (Hayward, CA, USA). Human factors Va, VIIa, IXa, X, Xa, prothrombin and thrombin were all from Haematologic Technologies (Burlington, VT, USA). Recombinant human factor VIII was from American Diagnostica Inc. Chromogenic substrates S-2765 and S-2238 were from Instrumentation Laboratory Company (MA, USA).

### 3.2. Protein Purification and Labeling

Bovine lactadherin was purified as previously described, and was labeled with Alexa Fluor 488 according to the package instructions. The ratio of fluorescein to lactadherin was 1.2/1 [30,31].

### 3.3. Preparation of RBCs and Treatment

The study was approved by the Ethics Committee of Harbin Medical University and consistent with the Helsinki Declaration. Peripheral vein blood was drawn from each eight healthy volunteers by antecubital venipuncture and anticoagulated with trisodium citrate 0.109 mol/L. These healthy donors were 19–22 years old, sex matched, non-smokers, and they had not taken any drugs for at least two weeks. RBCs were prepared within 30 min after blood collection by centrifugation for 15 min at 200 g at room temperature, and then the supernatant platelet-rich plasma (PRP) and leukocyte were removed carefully. IAA was diluted from a stock solution prepared in ethanol, and IS from a stock solution prepared in KCl.

Under a 5% CO_2_ humidified atmosphere at 37°C, RBCs (at a hematocrit of 0.4%) were maintained in Ringer solution (125 mM NaCl, 5 mM KCl, 1 mM MgSO_4_, 32 mM HEPES, 5 mM glucose and 1 mM CaCl_2_, pH 7.4) in 6-well culture plate and exposed to median and maximal uremic concentration of IS (0.1 mM and 1 mM) and IAA (20 μM and 50 μM) at indicated time points, respectively. Uremic solutes were compared with their respective controls (KCl or ethanol) diluted in the culture medium in the same range. In Ca^2+^-free Ringer solution, 1 mM CaCl_2_ was substituted by 1 mM glycol-bis (2-aminoethylether)-*N*,*N*,*N*′,*N*′-tetraacetic acid (EGTA).

### 3.4. Preparation of MDP and RMPs

For MDP preparation, PRP were centrifuged 20 min at 1500 g, and plasma was then harvested and centrifuged 2 min at 13,000 g to remove all residual platelets. The platelet free plasma (PFP) was centrifuged at 106,000 g for 1 h at 4 °C to remove MPs, aliquoted, and stored at −80 °C. RBCs in each well were collected by centrifugation (200 *g*, 15 min). The supernatants containing red blood cell-derived microparticles (RMPs) were harvested. As previously described [23], RMPs were pelleted by ultracentrifugation (106,000 *g*, 45 min, 4 °C) of the supernatants, and resuspended in 100 μL Ringer solution.

### 3.5. Flow Cytometry

To quantify PS exposure, RBCs suspended in Ringer solution were adjusted to 0.5–1 × 10^6^/mL, then 5 μL Alexa Fluor 488-conjugated lactadherin was added to the cell suspension respectively and incubated for 10 min at room temperature in the dark. Ten thousand events per sample were acquired and analyzed with BD FACS Diva Software. RMPs were defined as smaller than 1 μm and coexpression of lactadherin and CD235a. The number of RMPs per μL culture medium was calculated by Trucount Tube (with a precise number of fluorescent beads 48,678 to determine the number of MPs in a sample) after accumulation of 10,000 gated events and analyzed with Diva Software.

### 3.6. Confocal Microscopy

Observation of the PS exposure on RBCs was carried out as previously described [23]. RBCs were incubated with the indicated concentrations of Alexa Fluor 488-lactadherin for 10 min at room temperature in the dark, then washed to remove unbound dye and analyzed immediately. Images were obtained in LSM 510 SYSTEM (Carl Zeiss Jena GmbH, Jena, Germany).

### 3.7. Measurement of Intracellular Ca^2+^ Concentration

For detection of cytosolic Ca^2+^ concentration ([Ca^2+^]), each group of RBCs were washed in Ringer solution and then loaded with 2 μM Fluo 3/AM for 1 h at 37 °C in the dark. Subsequently, RBCs were washed twice and then resuspended in 200 μL Ringer solution to a final concentration of 1×10^6^/mL. Ca^2+^ dependent fluorescence intensity was measured and analyzed with BD FACS Diva Software.

### 3.8. Procoagulant Activity and Inhibition Assays

Procoagulant activity (PCA) of RBCs and MPs was evaluated by one-stage recalcification time assay in a KC4A-coagulometer (Amelung, Labcon, Heppenheim, Germany) [21]. 100 μL of RBC (1 × 10^8^) or MPs-containing suspension (prepared from 10 mL of the RBCs supernatants) was incubated with 100 μL of MDP at 37 °C. After 180 seconds, 100 μL of warmed 25 mM CaCl_2_ was added to start the reaction and the clotting time was recorded. All clotting assays were performed in triplicate. For the inhibition assay of coagulation time, 100 μL of RBCs or MPs suspensions were preincubated with 50 μL lactadherin (final concentration 128 nM) for 10 min at 37 °C. Clotting time was then recorded as above after addition of 100 μL MDP and 50 μL of warmed 50 mM CaCl_2_.

### 3.9. FXa and Prothrombinase Assays

For intrinsic factor Xa assay, a total of 10^5^ RBCs or 100 μL of RMPs-enriched suspension was incubated with 1 nM FIXa, 130 nM FX, 5 nM FVIII, 0.2 nM thrombin and 5 mM Ca^2+^ in FXa buffer (1 mL 10× TBS, 200 μL 10% BSA, 8.8 mL ddH_2_O) for 5 min at 25 °C. The reaction was stopped by addition of EDTA at a final concentration of 7 mM. After the addition of 10 μL S-2765 (0.8 mM) to each reaction, the quantity of FXa formed was determined immediately at 405 nm in kinetic mode on a Universal Microplate Spectrophotometer (PowerWave XS; Bio-Tek). Results were evaluated against the rate of substrate cleavage of a standard dilution of FXa. For the prothrombinase assay, RBCs were incubated with 1 nM FVa, 0.05 nM FXa, 1 μM prothrombin and 5 mM Ca^2+^ in prothrombinase buffer (1 mL 10× TBS, 50 μL 10% BSA, 8.95 mL ddH_2_O) for 5 min at 25 °C. EDTA and S-2238 were added to each microplate, and thrombin production was assessed as previously described [21]. Inhibition assays of coagulation complexes by lactadherin were performed as follows. RBC was incubated with 128 nM lactadherin for 10 min at 25 °C in Ringer solution. The quantity of thrombin or FXa formation was then assessed as above.

### 3.10. Statistical Analysis

Data are expressed as mean ± standard deviation (SD), and statistical analysis was made by Wilcoxon or Kruskal-Wallis test as appropriate. *p* < 0.05 was considered statistically significant.

## Supplementary Files

Supplementary File 1
