# Supplementary material for: Indolic Uremic Solutes Enhance Procoagulant Activity of Red Blood Cells through Phosphatidylserine Exposure and Microparticle Release
Source: Toxins (Basel). 2015 Oct 28;7(11):4390–403. doi: 10.3390/toxins7114390 (PMC4663509; doi:10.3390/toxins7114390)
Supplement: Supplementary File 1 [file toxins-07-04390-s001.pdf]

Supplementary Information

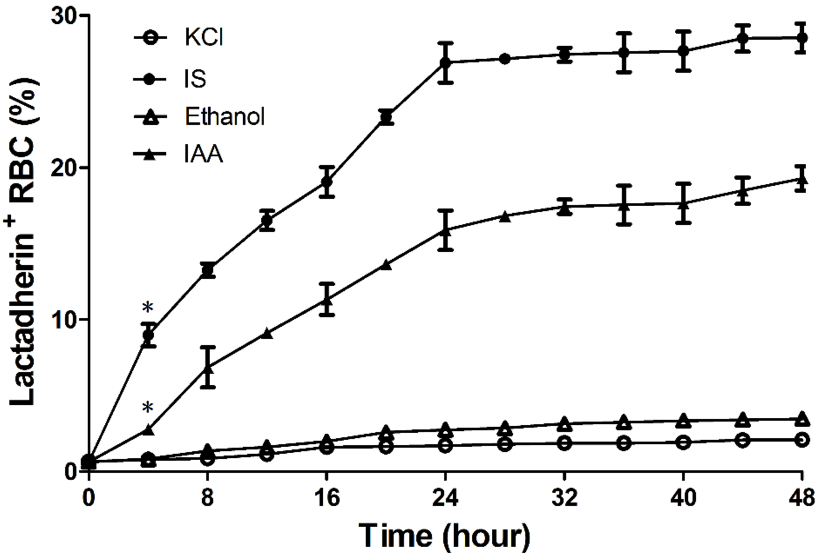

Figure S1. Kinetic analysis of PS exposure on RBC with different treatment. RBCs from healthy volunteers were incubated with IS (1 mM) and IAA (50 μM) for 48h, respectively. KCl or ethanol was utilized as their respective controls. At indicated time points (0, 4, 8, 12, 16, 20, 24, 28, 32, 36, 40, 48 h), lactadherin -binding percent of RBCs was evaluated by flow cytometry. Each point represents mean ± SD for four samples of independent experiments (* $p < 0.001$). PS, phosphatidylserine; IS, Indoxyl sulfate; IAA, indoxyl-3-acetate acid; RBC, red blood cell.
